# Crop Type Maps for Operational Global Agricultural Monitoring

**DOI:** 10.1038/s41597-023-02047-9

**Published:** 2023-03-28

**Authors:** Inbal Becker-Reshef, Brian Barker, Alyssa Whitcraft, Patricia Oliva, Kara Mobley, Christina Justice, Ritvik Sahajpal

**Affiliations:** 1 Department of Geographical Sciences, University of Maryland, College Park, MD 20742 USA; 2 GEOGLAM Secretariat, Geneva, Switzerland; 3 University of Strasbourg, The Engineering science, computer science and imaging laboratory (Icube), Strasbourg, France; 4 Universidad de Alcalá, Environmental Remote Sensing Research Group, Departamento de Geología, Geografia y Medio Ambiente, Alcalá de Henares, Spain; 5 Hémera Centro de Observación de la Tierra, Escuela de Ingeniería Forestal, Facultad de Ciencias, Ingeniería y Tecnología, Universidad Mayor, Santiago, Chile

**Keywords:** Environmental impact, Climate sciences

## Abstract

Crop type maps identify the spatial distribution of crop types and underpin a large range of agricultural monitoring applications ranging from early warning of crop shortfalls, crop condition assessments, production forecasts, and damage assessment from extreme weather, to agricultural statistics, agricultural insurance, and climate mitigation and adaptation decisions. Despite their importance, harmonized, up-to-date global crop type maps of the main food commodities do not exist to date. To address this critical data gap of global-scale consistent, up-to-date crop type maps, we harmonized 24 national and regional datasets from 21 sources covering 66 countries to develop a set of Best Available Crop Specific masks (BACS) over the major production and export countries for wheat, maize, rice, and soybeans, in the context of the G20 Global Agriculture Monitoring Program, GEOGLAM.

## Background & Summary

Ensuring food security is one of the major challenges we face in this century, especially in the face of a changing climate and a growing global population. With a rapidly growing demand for food, increasing conflicts, a highly interconnected global market, and increasingly extreme weather events, timely and accurate projections and estimates of global crop production are more important than ever^1^. Such estimates are a key component for well-functioning agricultural commodity markets and early warning and mitigation systems. One of the key international activities in support of transparent agricultural markets is the Group on Earth Observations Global Agriculture Monitoring (GEOGLAM) Crop Monitor for the G20 Agricultural Market Information System (AMIS) which provides a public good of open, timely, science-driven information on global crop conditions^2^. The AMIS and GEOGLAM initiatives were launched by the G20 Ministers of Agriculture following the food price crises in 2007/08 and 2010^3^. While the GEOGLAM initiative is focused on enhancing crop monitoring capabilities, in support of policies, investments, and decisions in food security and agricultural markets using satellite and *in situ* Earth observations (EO), AMIS provides an inter-agency platform of economists and policymakers who work together to enhance food market transparency and policy response for food security. Bringing together the principal trading countries of agricultural commodities, AMIS assesses global food supplies (focusing on wheat, maize, rice, and soybeans) and provides a platform to coordinate policy action in times of market uncertainty.

In support of these activities, AMIS requested that GEOGLAM develop monthly crop condition assessments likely to impact production for these four main commodity crops. Foundational in providing such information is the identification of where each crop of interest is growing. Together with crop calendars, crop type maps enable the extraction of crop specific signals from satellite data during the agricultural growing season that can track crop development through the season and forecast yields ahead of harvest^4,5^. Despite their high value for trade and food security assessments, within-season maps at a sufficiently granular resolution to enable field to global-scale analyses of crop condition and crop yield do not exist across all of the world’s agricultural areas^6,7^. While for years this dearth was owing at least in part to insufficient satellite data and limits on computational processing^8,9^, today the principal challenges are the lack of high-quality ground reference data for calibration and validation of crop classifications^10,11^. Nevertheless, a range of crop type map products derived from satellite imagery does exist at national and regional scales (e.g.^12,13^). In addition, at the global scale, there are products such as the IFPRI SPAM-2010^14^, M3-Crops^15^, and MIRCA2000^16^ that provide information on crop type distribution based on sub-national statistics and a spatial allocation model at the 10Km resolution. While these represent the current state of the art for global crop type distribution, they are based on spatial models and subnational statistics rather than the spectral signal of a crop and they are at a very coarse resolution (10 km) and are out of date (i.e. represent croplands circa 2010). In short, they may represent the national or subnational total land area of each crop, though the spatial location of crops may not be correct, which presents a critical issue for their application in masking for within-season crop monitoring.

To meet the needs of the GEOGLAM Crop Monitor to accurately mask crop type with as up-to-date information as available, we developed a harmonized global set of crop specific maps for the four major grains (wheat, maize, rice, and soybeans) following an exhaustive identification and collection of the most recent, highest quality existing crop type maps at national and regional sources. Similar to the efforts by Fritz *et al*.^17^ and Waldner *et al*.^18^ that created a unified general cropland product based on existing cropland products, we designed a criteria system to assess the best data sets with regards to timeliness, accuracy, spatial resolution, and data source. The result is the first set of global crop type maps, at the 0.05 degree resolution, derived from satellite imagery, covering the major producer and export countries for the four main crops, referred to herein as the GEOGLAM Global Best Available Crop Specific Masks (GEOGLAM-BACS). These maps are used operationally within the GEOGLAM Crop Monitor in the creation of monthly global crop condition assessments and are updated on an annual basis as new crop type maps become available. The dataset is made publicly available with this publication on CropMonitor.org as well as on Zenodo and at the time of submission refers to version v.1.0.

## Methods

Several steps were required for developing the GEOGLAM-BACS. These included the following, which are further detailed below:

- Dataset collection and selection of a base layer
- Criteria evaluation and scoring
- Product selection based on scores
- Generation of a unified map (updated as new data become available)

### Data set selection

The Group on Earth Observation Global Agriculture Monitoring (GEOGLAM) required global crop-specific masks to provide accurate crop assessments for the primary crops (maize, wheat, soybean, and rice) that AMIS (Agricultural Market Information System) is focused on. In response to this requirement and acknowledging the need to ensure the spatial fidelity of crop locations while building upon years of community product development, we undertook a study of existing global, regional, and national crop-specific map products through exchanges with the various national and regional focal points that contribute to the Crop Monitor for AMIS^2^, the GEOGLAM expert networks, and literature reviews. Three gridded products based on different spatial allocation models and subnational statistics were identified at the global scale (SPAM, MIRCA, and M3), but the Spatial Allocation Production Model 2010 (SPAM-2010 physical area layer was selected as the foundation because it provides a gridded product containing the physical area of the GEOGLAM target crops at a spatial resolution of 10 × 10 km^19,20^. However, the SPAM-2010 physical area crop mask refers to data from 2010 or thereabout, rendering it out of date. Further, it is based on a statistical allocation model that evenly distributes the crop physical area density across croplands based upon the administrative level for which the statistics were obtained, and therefore can result in inaccurate locations of crops. Given the vital importance of accurate cropping locations, solely relying upon SPAM-2010 to mask current croplands is insufficient for remote sensing-based analyses.

**Fig. 1:**
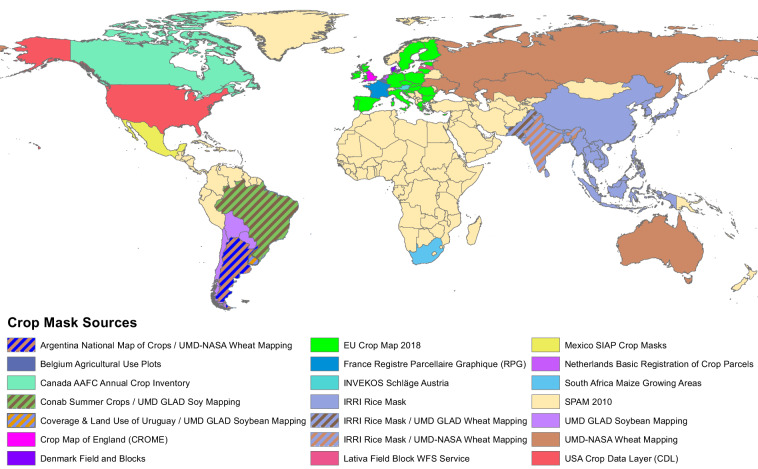
Individual products used per country to assemble the final GEOGLAM Best Available Crop Masks.

Due to these features coupled with the ongoing mandate of the GEOGLAM Crop Monitor to report on current-season crop conditions, we needed to derive an updated product based on an updateable method for generating timely and accurate maps of major crops. The crop masks incorporated into GEOGLAM-BACS masks were either produced by GEOGLAM partners from moderate-resolution (10–250 meter) satellite data or generated by Agricultural Ministries or Councils of some countries (Table 8, Fig. 1). They therefore, represent the most up-to-date crop masks available at the time of submission. Additionally, the GEOGLAM-BACS masks used national crop production information and expert knowledge to refine the crop area of some countries, bringing high-value expert input from many corners of the globe into a unified product. The top five global producers of each commodity (Table 1) were prioritized as those countries account for between 82–97% of global crop production. The GEOGLAM-BACS integrates 16 out of the 20 crop-type masks for these top producers.

**Table 1:** Top five producers for each crop using the 5-year mean production value from 2017–2021 according to statistics from USDA PSD Online (accessed 10/20/2022).

Wheat	Maize	Rice	Soybean
*China*	United States	China	Brazil
European Union	*China*	India	United States
India	Brazil	Indonesia	Argentina
Russia	European Union	Bangladesh	*China*
United States	Argentina	Vietnam	*India*

Countries in italics indicate where updated crop-specific national masks were not publicly accessible.

Several criteria were used to determine a product’s fitness for inclusion within GEOGLAM-BACS and are described below. In countries where no specific crop type maps were available, the SPAM 2010 data remains.

### Selection criteria and scoring

Similar to the approach described by Waldner *et al*.^21^, five criteria were considered to guide the selection and evaluation of the crop specific maps. These consisted of seasonal specificity (SS), timeliness (Ti), spatial resolution (SR), accuracy (Ac), and data source (DS) (Tables 2–6). Each criterion has a range of scores with 4 or 3 as the highest score (depending on the criterion) and 1 as the lowest.

**Table 2:** Rules for the seasonal specificity criteria, where the highest score of 3 indicates all seasons captured while the lowest score of 1 means all seasons are aggregated into 1 mapped layer.

Rules for seasonal specificity criteria	
Criteria	Score
All seasons are represented as unique layers	3
One of the multiple seasons represented	2
Multiple seasons aggregated	1

**Table 3:** Rules for the timeliness criteria, where the highest score of 4 indicates an in-season product and the lowest score of 1 indicates a crop map older than 3 years.

Rules for timeliness	
Criteria	Score
Current Season (In-Season)	4
Post Harvest/End of Season (within 1 year)	3
Recent (within 1 to 3 years)	2
Out-of-date (older than 3 years)	1

**Table 4:** Rules for the spatial resolution criteria, where the highest score of 4 indicates a high-resolution product of 5–20 meters, while a score of 1 indicates a very course resolution product of >500 meters.

Rules for spatial resolution	
Criteria	Score
High (5–20 meter)	4
Moderate (20–100 meter)	3
Coarse (100–500 meter)	2
Very Coarse (>500 meter)	1

**Table 5:** Rules for the accuracy criteria, where the highest score of 4 indicates a high accuracy of >90% overall, while a score of 1 indicates that the accuracy of the product is not reported.

Rules for accuracy	
Criteria	Score
High (>90% overall accuracy)	4
Good (80–90% overall accuracy)	3
Low (<80% overall accuracy)	2
Not Reported	1

**Table 6:** Rules for the data source criteria, where the highest score of 3 indicates a national-level product, while a score of 1 indicates a global-level product.

Rules for data source	
Criteria	Score
National	3
Regional	2
Global	1

#### Seasonal specificity criteria

A key element for in-season crop assessments and yield forecasting is accounting for multiple growing seasons. As such, a requirement for the GEOGLAM-BACS was to account separately for winter wheat and spring wheat, for the first and second maize seasons, for the first and second soybean seasons, and for multiple rice growing seasons (up to three seasons). The Seasonal Specificity (SS) Criteria was designed to capture this information, where a score of 3 indicates all seasons captured, a score of 2 indicates that one of the multiple seasons is captured, and a score of 1 means all seasons are aggregated into 1 mapped layer (i.e. winter wheat and spring wheat are not captured separately, but rather are aggregated into one class of wheat) (Table 2).

#### Timeliness (Ti) criterion

In most growing regions of the world, croplands are dynamic with changes in planting occurring from one season to the next due to crop rotation, weather, agricultural markets, and policies. As such, a within-season crop-specific map at field scale would be ideal^6,22–25^. While there is a large push from the community to produce such products at scale (i.e.^10,11^), to date, such products are not widely available. Nevertheless, broadly speaking a recent season is generally more likely to represent crop specific extent relative to older products, especially at coarse resolution per pixel percent crop type maps such as the BACS product^22^. As such, the timeliness criterion is designed to give the highest score (4) to within-season products and the lowest score (1) to the out-of-date products defined as older than 5 years (Table 3). It is worth noting here that none of the crop products in BACS had a score of 4.

#### Spatial resolution criterion

A range of studies have characterized in detail the spatial resolution requirements for accurately mapping crop types, which account not only for field size but also account for field shapes, crop diversity, and overall landscape complexity^26–28^. However, for the GEOGLAM-BACS products, we took a simplified approach and considered that the higher spatial resolution products would generally be able to characterize crop types better than the coarser resolution products. While we acknowledge that higher spatial resolution does not always translate to higher accuracy, they do often perform better when produced with local training and validation datasets which is the case for the higher-resolution products integrated into the GEOGLAM-BACS dataset.

As such, the higher-resolution products (5–20 m) received the highest score (4) and the lowest-resolution products (>500 m) received the lowest score (1) (Table 4). The four resolution classes were defined based on spatial resolution requirements as articulated by the GEOGLAM community and defined in^9^.

#### Accuracy criterion

The accuracy criterion scoring was based on the accuracy metrics available for each of the crop type products (Table 5). Where not available, scoring is based on an assessment against sub-national level published statistics. This criterion was based on four categories, with the above 90% reported overall accuracy score receiving a score of 4 and overall accuracy of less than 80% received a score of 2. Several of the products did not report accuracy at all, or did not report accuracy for a specific crop type or country, and therefore where possible we approximated the accuracy of these products based on a comparison with subnational-level official statistics. We recognize the issues with this approach, but given that we did not have any other data on their accuracy this was an acceptable approximation for this purpose. Where the accuracy of these products could not be discerned for these purposes, they received the lowest score (1).

#### Data source criterion

This criterion aims to capture the relative specificity of the products for the countries they cover. Generally speaking, products that are developed at a sub-national to national scale are based on models and ground data collected for that specific country and are specialized for that country. At the regional level, the mapped products and models are usually more generalized to map larger regions, and generally speaking have less national-specific data, and lower accuracies at the national scale. Likewise, products that are at the global scale, often rely on generalizations developed in locations with ground data and often extrapolate to map regions with little or no ground data. While great progress is being made in developing robust and scalable models utilizing sparse ground data sets^10,11,29–32^ still, for the most part, the sub-national and national-level products tend to have higher accuracies than do regional and global data. As such in this criterion, the national products received the highest score (3) and the global products received the lowest score (1) (Table 6).

### Scoring & product selection

The scores for each criterion were then aggregated to compute the fitness indicator (FI) as follows:

$${\rm{FI}}={\rm{SS}}+{\rm{Ti}}+{\rm{SR}}+{\rm{Ac}}+{\rm{DS}}$$

Where FI is the Fitness Indicator, SS is the seasonal specificity score, Ti is the timeliness score, SR is the spatial resolution score and Ac is the accuracy score, and DS is the data source score (Table 7).

**Table 7:** Crop Masks product scoring based upon the defined selection criteria.

Country/Region of Coverage	Product	Seasonal Specificity	Timeliness	Spatial Resolution	Accuracy	Data Source	TOTAL
Argentina	National Map of Crops 2018/2019 Campaign	1	3	4	3	3	14
Argentina	UMD/NASA Winter Wheat Mapping	3	1	2	4	3	13
Asia	IRRI Rice Mask	1	1	2	3	2	9
Australia	UMD/NASA Winter Wheat Mapping	3	1	2	4	3	13
Austria	INVEKOS Schläge Austria 2019	3	2	4	4	3	16
Belgium	Agricultural use plots ALV 2016, Anonymous Agricultural Plot (2018) (PAA)	3	1	4	4	3	15
Brazil	UMD GLAD Brazil Soybeans	3	1	3	3	3	13
Brazil	Conab Crop Areas	3	2	3	3	3	14
Canada	AAFC Canada Annual Crop Inventory	3	3	4	3	3	16
Denmark	Fields and Blocks	3	2	4	1	3	13
England	Crop Map of England (CROME) 2019	3	2	3	1	3	12
European Union	EU Crop Map 2018	3	2	4	3	2	14
France	France Registre Parcellaire Graphique (RPG)	3	2	4	4	3	16
Global	SPAM 2010	1	1	1	1	1	5
India	UMD/NASA Winter Wheat Mapping	3	1	2	4	3	13
Kazakhstan	UMD/NASA Spring Wheat Mapping	3	1	2	4	3	13
Latvia	Field Block WFS service	3	2	4	1	3	13
Mexico	Mexico SIAP Crop Mask	3	1	4	2	3	13
Netherlands	Basic Registration of Crop Parcels (BRP) 2019	3	2	4	4	3	16
Pakistan	UMD GLAD Pakistan Winter Wheat	3	1	3	3	3	13
Russian Federation	UMD/NASA Winter Wheat Mapping	2	2	2	4	3	13
South Africa	Maize growing areas	3	1	4	4	3	15
Ukraine	UMD/NASA Winter Wheat Mapping	3	2	2	3	3	13
Uruguay	Integrated Map of Coverage/Land Use of Uruguay	3	1	4	4	3	15
USA	USDA NASS CDL	3	3	4	3	3	16

Full references for each Country/Region of Coverage associated Product are included with annotation in the References section. References for Denmark, Latvia, Mexico, and South Africa are not included as the products are not publicly accessible.

### Overview of scores

**Fig. 2:**
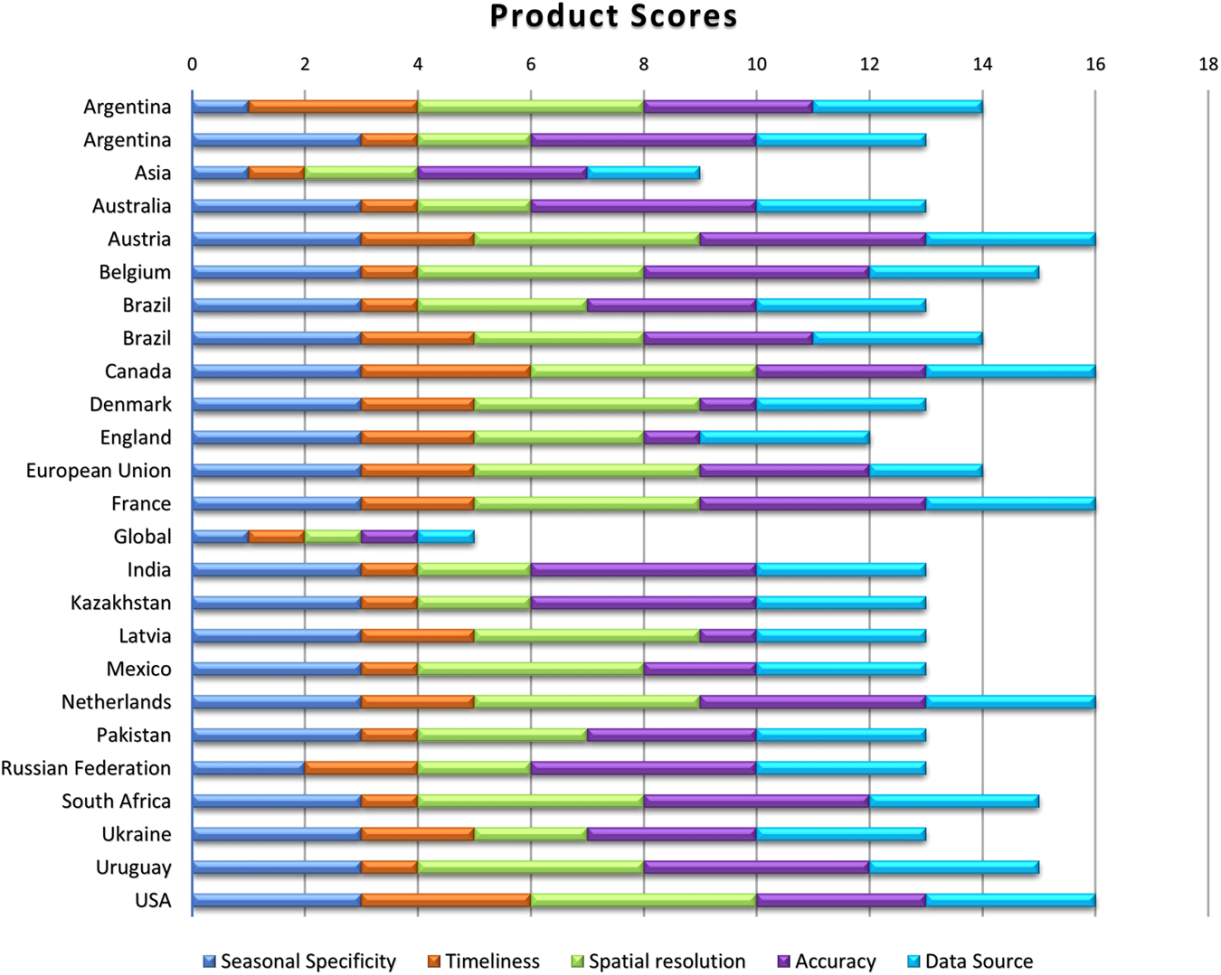
Cumulative scores and the contribution from each criterion for all of the individual products used to assemble the final GEOGLAM Best Available Crop Masks.

The highest score possible accounting for the highest rank in each category is 18. While none of the products received this score (as none are in-season products at 10-meter resolution), several products received high scores, with eleven products scoring between 16 and 14, twelve products scoring between 13 and 11, one scoring 9, and one scoring 5 (the initial base map) (Fig. 2). This highlights one of the most pressing current gaps in crop specific maps, which is a lack of in-season crop type masks that are especially crucial for tracking specific crop development, input deficiencies, yield forecasting, planted area, etc. during the growing season itself. While many of the products were recent (within the past 1 to 3 years), a large portion of the major staple crop-producing regions of the world do not have publicly available recent crop type maps, notable countries are India and China, two of the world’s largest crop producers and consumers. An area of low scores is for SS in countries where there is more than one season per year for the same crop such as summer vs. spring-planted maize or winter vs. spring wheat. An important example of this is the differentiation between spring wheat and winter wheat in Russia or early and late planted soybeans in Argentina.

### Generation of unified map

As the collection of national, regional, and global crop-specific masks are at a variety of spatial resolutions, all the masks were harmonized to a common resolution. The 0.05-degree resolution of the climate modeling grid (CMG) MODIS products was selected for the standardized product. This resolution was chosen to match the wide variety of climate datasets available at the CMG level for agricultural monitoring. Additionally, it has been shown that when detailed in-season crop masks are not available over areas with crop rotations, a coarse resolution mask, such as the 0.05-degree CMG, can be used to accurately monitor and yield forecast for specific crops using Normalized Difference Vegetation Index (NDVI) time-series data^22^.

The crop-specific SPAM 2010 products were converted to percent per crop, per pixel, and then resampled to CMG level from the original 10 × 10 km grid. Since winter and spring wheat have different growing calendars and require detailed monitoring during different periods, the SPAM-2010 v2 wheat layer was split out into separate seasonal masks. In locations where seasonal-specific crop masks could be obtained (Canada, United States, Russia winter wheat, and Kazakhstan spring wheat), they were used. For the remaining locations and seasons (Russia spring wheat, China winter and spring wheat, and Kazakhstan winter wheat), sub-national sown area statistics were used to identify the percent of the SPAM-2010 v2 wheat layer associated with each season. This was done by incrementally reducing the percent per pixel value across each sub-national region uniformly until the area of the crop mask within the sub-national region matched the reported sown area for that region.

**Fig. 3:**
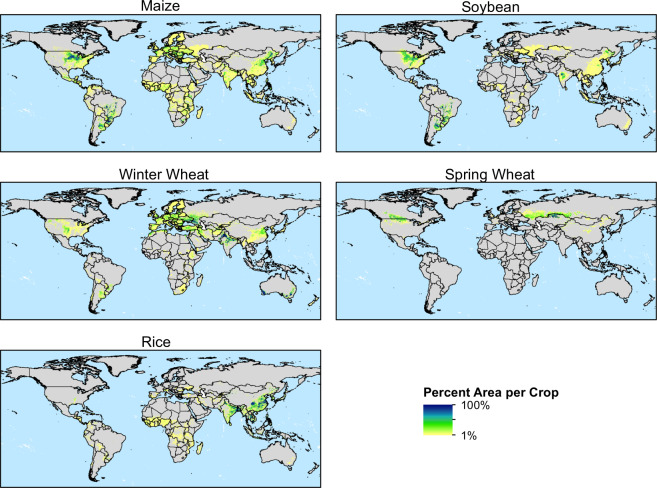
All five crop masks are shown as a percent per pixel at 0.05 degrees that are covered by an individual crop. Areas with a higher percent per pixel of a crop are shown in darker blue while areas with a lower percent per pixel are shown in light yellow.

**Fig. 4:**
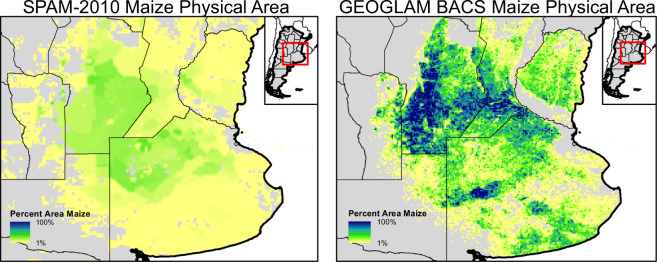
SPAM 2010 v2.0 physical area for maize in Argentina at CMG level (left) compared to the physical area for maize from the Argentina National Map of Crops 2018/2019 campaign at CMG level (right).

The crop-specific masks used in assembling the GEOGLAM-BACS masks came in a variety of formats and spatial resolutions. The steps used to derive the final standardized format and spatial resolution vary depending on the source. Generally, each of the designated crops was separated and converted over to a raster grid at its native spatial resolution. Then each crop type gridded layer was converted to a binary grid of “1” for the crop and “0” for no-crop. Using the CMG grid, each CMG cell was then overlaid on top of the crop-specific raster where the number of crop cells was divided by the total number of cells covered by the CMG cell to get the percentage of the crop within per pixel. The final product was then assembled by replacing the data from within the resampled SPAM-2010 product where the national and regional products were found to be available (Figs. 3, 4).

## Data Records

The GEOGLAM-BACS masks are a collection of five global Geotiff files containing flat rasters at 0.05 degrees, one for each of the AMIS crops (Maize, Soybean, Rice, Winter Wheat, and Spring Wheat). A comprehensive metadata file accompanies the rasters. Values represent the percent of the specific crop per pixel, ranging from 0 to 100%. Within the rasters, values were converted to integers whereby the values are from 0 to 10,000, where 10,000 is equal to 100.00%. This was done to preserve small variations across the landscape of less than 1% while reducing the size of the dataset. The GEOGLAM-BACS will be updated regularly as new and more recent products become available. The updated rasters and metadata files will be pushed to the Zenodo repository as well as hosted on www.CropMonitor.org.

For those countries and regions that have had the original SPAM 2010 v2 data replaced, Table 8 identifies the specific products used. The data is accessible on the Zenodo platform^33^: https://zenodo.org/record/7230863#.Y1F3mnZKh9M.

**Table 8:** List of all crop mask products used in the creation of the GEOGLAM Best Available Crop Specific Masks (BACS).

Country/Region of Coverage	Product	Crop(s) covered	Native Resolution	Season Coverage	References
Argentina	National Map of Crops 2018/2019 campaign	Maize, Soybean	30 meter	2018–2019	^41^
Argentina	UMD/NASA Wheat Mapping	Winter Wheat	500 meter	2015	^42^
Asia	IRRI Rice Mask	Rice	500 meter	2000–2003	^43–46^
Australia	UMD/NASA Wheat Mapping	Winter Wheat	500 meter	2014–2018 avg	^42^
Austria	INVEKOS Schläge Austria 2019	Maize, Soybean, Winter Wheat, Spring Wheat	Field Scale	2019	^47^
Belgium	Agricultural use plots ALV 2016, Anonymous agricultural plot (2018) (PAA)	Maize, Soybean, Winter Wheat, Spring Wheat	Field Scale	2018, 2016	^48^
Brazil	Conab summer crop areas	Maize, Rice, Winter Wheat	Field Scale to 250 m	2014–2021	^49^
Argentina, Brazil, Bolivia, Chile, Paraguay, Uruguay	UMD GLAD Annual Soybeans	Soybean	30 meter	2020	^50,51^
Canada	AAFC Canada Annual Crop Inventory	Maize, Soybean, Winter Wheat, Spring Wheat	30 meter	2019	^52^
Denmark	Fields and Blocks	Maize, Winter Wheat, Spring Wheat	Field Scale	2019	N/A (not publicly accessible)
England	Crop Map of England (CROME) 2019	Maize, Soybean, Winter Wheat, Spring Wheat	64 meter	2019	^53^
European Union	EU Crop Map 2018	Winter wheat, Maize	10 meter	2018	^54^
France	France Registre Parcellaire Graphique (RPG)	Maize, Soybean, Rice, Winter Wheat, Spring Wheat	Field Scale	2018	^55^
Global	SPAM 2010 v2.0	Maize, Soybean, Rice, Wheat	0.083 degrees	2010	^56^
India	UMD/NASA Wheat Mapping	Winter Wheat	500 meter	2014	^42^
Kazakhstan	UMD/NASA Wheat Mapping	Spring Wheat	500 meter	2015	^42^
Latvia	Field Block WFS service	Maize, Soybean, Winter Wheat, Spring Wheat	Field Scale	2019	N/A (not publicly accessible)
Mexico	Mexico SIAP crop mask	Maize, Winter Wheat, Spring Wheat	Field Scale	2019	N/A (not publicly accessible)
Netherlands	Basic Registration of Crop Parcels (BRP) 2019	Maize, Soybean, Winter Wheat, Spring Wheat	Field Scale	2019	^57^
Pakistan	UMD GLAD Pakistan Winter Wheat	Winter Wheat	250 meter	2014	^58^
Russian Federation	UMD/NASA Wheat Mapping	Winter Wheat	500 meter	2019	^42^
South Africa	Maize growing areas	Maize	Field Scale	2014	N/A (not publicly accessible)
Ukraine	UMD/NASA Wheat Mapping	Winter Wheat	500 meter	2019	^42^
Uruguay	Integrated Map of Coverage/Land Use of Uruguay	Rice	10 meter	2018	^59^
USA	USDA NASS CDL	Maize, Soybean, Rice, Winter Wheat, Spring Wheat	30 meter	2019	^60,61^

Shortened references for each Country/Region of Coverage and associated Product are included in the “References” column. References for Denmark, Latvia, Mexico, and South Africa are not included as the products are not publicly accessible at their native resolution.

## Technical Validation

The majority of the crop specific masks used in the creation of the GEOGLAM-BACS have associated validation and accuracy metrics that are publicly available (Table 9). Similar to work done by Pittman *et al*.^34^ and Pérez-Hoyos *et al*.^35^, the crop masks that do not have publicly available validation information were validated against reported sub-national sown area statistics for the specific reference year (Table 10). While we recognize there are many limitations to such an approach to validation, this was the only feasible way for providing some validation of these products. The comparisons against sub-national sown area statistics are provided in Table 10.

**Table 9:** Crop Mask products with validation already.

Country/Region of Coverage	Product	Validation Results	References
Asia	IRRI Rice Mask	1. South Asia: Accuracies from 67% to 100% for individual rice classes, with an overall accuracy of 80% for all classes	^43–46^
		2. Bangladesh: Rice versus non-rice exceeded 90% accuracy in all three seasons, and the accuracy of the five rice classes varied from 78% to 90% across the three seasons.	
		3. Nepal: Fuzzy classification accuracies range between 67% and 91% for various rice classes, with an accuracy of 82% for field-plot data.	
		4. South and Southeast Asia: Area estimates of paddy rice were highly correlated at the national level and positively correlated at the subnational levels, although the agreement at the national level was much stronger.	
Argentina, Brazil, Bolivia, Chile, Paraguay, Uruguay	UMD GLAD South America Soybeans	Overall soybean map accuracy ranges from 94–96%	^50,51^
Argentina	National Map of Crops 2018/2019 campaign	For the seven growing seasons considered, overall accuracies were higher than 85% and, in most years, higher than 90%.	^41^
Canada	AAFC Canada annual Crop inventory	The overall accuracy of Canadian crop inventory maps is 85 percent at the national scale but varies based on crop type, region, and year.	^52^
European Union	EU Crop Map 2018	The overall accuracy of the map is 80.3% when grouping main crop classes and 76% when considering all 19 crop type classes separately. Individual crop accuracies vary.	^54^
Global	SPAM 2010	N/A	^56^
England	Crop Map of England (CROME) 2019	The results were checked against survey data collected by field inspectors and visually validated.	^53^
Pakistan	UMD GLAD Pakistan Winter Wheat	While there wasn’t validation data for the crop type map itself, it was used as input for yield forecasts where forecasts for the 2007/2008 to 2012/2013 growing seasons were within 0.2% and 11.5% of final reported values in Punjab Province (most important wheat producing region in Pakistan).	^58^
USA	USDA NASS CDL	The most recent 2019 CDL achieves an 83 percent overall accuracy for all crops at the national scale. In general, the accuracy of the large area row crops ranges from 80 to 90 percent.	^60,61^

Shortened references for each Country/Region of Coverage and associated Product are included in the “References” column. Full references for each Country/Region of Coverage and associated Product are included with annotation in the References section.

**Table 10:** Comparison of crop masks that lack published validation information with sub-national sown area statistics from the crop mask reference year.

Country	Winter Wheat	Spring Wheat	Maize	Soybean	Rice
**Brazil**	0.9977	N/A	0.8275	N/A	0.8834
**Mexico**	0.85	0.643	0.657	N/A	N/A
**South Africa**	N/A	N/A	0.9104	N/A	N/A
**Austria**	0.998	*	0.958	0.9543	N/A
**Belgium**	0.986	*	0.984	!	N/A
**Denmark**	*	*	*	N/A	N/A
**France**	0.944	*	0.9976	!	1
**Netherlands**	0.9826	*	0.9913	*	N/A
**Uruguay**	N/A	N/A	N/A	N/A	0.9995
**Latvia**	*	*	*	!	N/A

R-squared values for each crop per country, each country, and by each crop.

*Only national level data available for the year in question.   ! No official statistics available.

N/A Crop was not included in the masks.

### Known issues & required improvements

GEOGLAM-BACS, as indicated by its name, represents the best-available, most up-to-date globally harmonized dataset on the locations of the four major commodity crops wheat, maize, rice, and soybean. Still, there remain gaps as indicated by this technical validation, and enumerated briefly below:

- While we have made our best effort to validate GEOGLAM-BACS, in many areas validation data for specific locations simply does not exist. All crop monitoring activities, including that of the GEOGLAM Crop Monitor, would be strengthened with quantitative validation that would empower more precise and risk-informed decision-making^36^. Critical to this effort is expanding the collection and availability of ground data for training and validation. Advances have been made in generating and interpreting labeled datasets to assist in these efforts (e.g.^31,32,37^), and should be further developed, particularly in data-sparse regions which often correlate with food insecurity^38^.
- The GEOGLAM-BACS are not currently produced in-season. This is a major priority for monitoring crops as they develop throughout the growing season, yield forecasting, producing within-season cropped area estimates, damage estimates from extreme weather, conflict, etc. Relying on prior year information is the state of the science at global scale^22^, however with advances in  public and commercial satellite missions, that together deliver sub-weekly or even daily observations at 3–30 m (e.g. Landsat 8, Landsat 9, Sentinel1 & 2, Planet Doves;^9,39^, and advances in cloud compute, ML, and digital data collection, within-season crop type mapping is within sight and has been articulated as a priority for GEOGLAM Essential Agriculture Variables (EAVs)^25^ and the European Space Agency’s WorldCereal project^29,40^.
- In areas of the world where multiple seasons of the same crop exist – for example, maize and rice – in some cases seasonal specificity is lacking in the current GEOGLAM-BACS. Complementing crop type mapping with crop type calendar information is an important research area(e.g.^62^, again prioritized by the GEOGLAM EAVs.
- There remain areas for which no recent satellite based crop type data exists, and the product relies on SPAM 2010’s statistical allocation model. It is unclear whether these locations are representative of true crop locations, and merit prioritization in future satellite-mapping activities.
- Of particular importance to human livelihoods is the improvement of information in areas with acute food insecurity and/or characterized by smallholder agriculture. This requires fine spatial resolution data, which as mentioned is now available between public and private optical and synthetic aperture radar missions, as well as appropriate ground data, which is often the primary bottle neck.

As demonstrated by GEOGLAM-BACS, one of the most powerful tools in advancing global agricultural monitoring is international collaboration. The GEOGLAM-BACS is a public good that would not be possible without the inputs and substasive contributions of GEOGLAM partners around the world.

## Usage Notes

The primary use of this dataset is for underpinning EO-based, global, crop-specific condition monitoring for four of the major crop commodities in the major production/exporting countries, through the GEOGLAM Crop Monitor activity. As noted in the prior section (Technical Validation: Known Issues & Required Improvements), there is room for improvement concerning validation, extent, resolution, and seasonal specificity, and these limitations should be noted in the application of these data in different monitoring activities. Nevertheless, the data are already being operationally used within the GEOGLAM Crop Monitor Reports.

## Data Availability

No custom code was used to generate or process the GEOGLAM-BACS masks. The software used in the assembly of the masks was ArcMap version 10.6.
